# PD-L1 correlated gene expression profiles and tumor infiltrating lymphocytes in pancreatic cancer

**DOI:** 10.7150/ijms.61771

**Published:** 2021-07-05

**Authors:** Jiajin Li, Lu Yin, Yumei Chen, Shuxian An, Yi Xiong, Gang Huang, Jianjun Liu

**Affiliations:** 1 Department of Nuclear Medicine, Renji Hospital, School of Medicine, Shanghai Jiao Tong University, Shanghai 200127, China.; 2 Institute of Clinical Nuclear Medicine, Renji Hospital, School of Medicine, Shanghai Jiao Tong University, Shanghai 200127, China.; 3 Department of Cardiology, Gongli Hospital of Shanghai Pudong New Area, Shanghai 200135, China.; 4 Department of Bioinformatics and Biostatistics, School of Life Science and Technology, Shanghai Jiao Tong University, Shanghai 200240, China.; 5 Shanghai Key Laboratory of Molecular Imaging, Shanghai University of Medicine and Health Sciences, Shanghai 201318, China.

**Keywords:** PD-L1, Prognostic value, Pancreatic cancer, TCGA, GSEA

## Abstract

**Objective:** To study the expression and clinical value of PD-L1 gene in pancreatic cancer, and to predict the role of PD-L1 gene in the development of pancreatic cancer.

**Methods:** The pancreatic cancer datasets were downloaded from the Cancer Genome Atlas (TCGA) and the Oncomine to obtain the PD-L1 gene expression profile and clinical information. Bioinformatics methods were used to analyze the correlation between the expression level of PD-L1 gene in pancreatic cancer and clinicopathological indicators, as well as its influence on prognosis. GSEA and WGCNA analysis was performed to predict the possible pathways of PD-L1 gene regulation in pancreatic cancer. TIMER and MCP-counter were used for PD-L1 with immune infiltration. The genes interact with PD-L1 were also investigated by STING and immunoco-precipitation combined with mass spectrometry analysis (IP-MS).

**Results:** In TCGA database, the overall survival of patients with high expression of PD-L1 gene was significantly lower than that of patients with low expression of PD-L1 gene (χ^2^ = 12.52, P < 0.001). The samples with high expression of PD-L1 gene showed enrichment of 8 pathways including toll-like receptor signaling pathway and NOD receptor signaling pathway (P < 0.01, FDR < 0.05). Immune infiltration analysis suggested that PD-L1 were associated with monocytic lineage (r = 0.5). The proteins interacting with PD-L1 are mainly concentrated in RNA binding, ribosome, spliceosome and other biological processes or pathways.

**Conclusion:** PD-L1 gene may play an important role in the development of pancreatic cancer and is expected to be a prognostic indicator of pancreatic cancer.

## Introduction

Pancreatic cancer is a highly malignant tumor of the digestive tract. There was about 496,000 new cases and 466,000 deaths worldwide in 2020 1. The 5-year survival rate of pancreatic cancer was merely 9%, which made it became one of the worst prognosis malignant tumors 2. About 90% of pancreatic cancer is ductal adenocarcinoma originating from the glandular epithelium, which is difficult to diagnose and treat 3. Due to the low rate of surgical resection and poor efficacy of chemotherapy for pancreatic cancer, there is still a lack of ideal treatment methods at present 4.

Programmed Death Ligand 1 (PD-L1) immunotherapy has been successful in the treatment of lung cancer, urothelial carcinoma and other tumors, bringing hope for the treatment of pancreatic cancer 5. However, a series of problems, such as drug resistance, patient screening and combination therapy selection, limit the clinical application of PD-L1 immunotherapy in pancreatic cancer. To resolved these problems, it is required that the comprehensive understanding of specific mechanisms underlying PD-L1 regulation in pancreatic cancer.

Programmed death ligand 1 (PD-L1) is a 40 kDa transmembrane protein, encoded by CD274 gene. The expression of PD-L1 can be induced in tumor and other tissues by inflammatory factors. When activated, PD-L1 suppresses the immune system in cancer, pregnancy, tissue transplantation, and autoimmune diseases 6.At present, the correlation between PD-L1 gene and pancreatic cancer still needs to be further studied.

This study intends to explore the expression of PD-L1 gene in pancreatic cancer tissue and its clinical significance based on the Cancer Genome Atlas (TCGA) and Oncomine database. In addition, we will also analyze the relationship between PD-L1 and immune cell infiltration. Potential PD-L1 related proteins and pathways were also analyzed in this study.

## Methods

### Patient information

The RNA-sequencing data and corresponding patient clinical information of 177 pancreatic cancer tissue samples were downloaded from the TCGA database (https://cancergenome.nih.gov/), among which 169 patients had matching mRNA expression profiles and survival data.

### Data processing and patient grouping

Download package edge R (http: //www. bioconductor. Org/packages/release/bioc/HTML/edgeR. HTML), for mRNA raw data based on weighted average truncated M value standardization, and the log2 transformed for subsequent analysis. Based on the expression profile of PD-L1 gene and follow-up data of pancreatic cancer patients, the optimal block cutoff value was determined according to X-tile 3.6.1 software (Yale University School of Medicine, New Haven, CT, USA). For the TCGA datasets, the optimal cutoff value of 5.93 was divided into the high expression group (20 cases) and the low expression group (149 cases). For the Oncomine datasets, the optimal cutoff value of 0.71 was divided into the high expression group (12 cases) and the low expression group (13 cases).

### Enrichment analysis of GSEA

Gene Set Enrichment Analysis (GSEA) software was used to study the correlation between PD-L1 gene expression level and gene sets in Kyoto Gene and Genome Encyclopedia pathway^7^. A continuous phenotype was established according to the expression level of PD-L1 genes. The Pearson correlation coefficient between PD-L1 and the reference gene set was calculated by the default weighted enrichment statistic, and gene sequencing was conducted according to the correlation coefficient. The number of random combinations was set to 1000, and the normallized enrichment score (ES) and normallized enrichment score (NES) were calculated, with standardized P < 0.01 and false discovery rates (FDR) < 0. 05 as significant enrichment.

### Co-expression gene analysis

The co-expression gene and enrichment analysis were applied to WGCNA package of R software, which revealed the correlation between genes 8. Because the genes with little expression variation usually represent noise, we filter the most variable genes (SD > 1.2) and construct a network. The power of β was set to 5 to ensure a scale-free network. The minimum number of module genes was set to 30. The hierarchical cluster tree summarizes the gene modules of different colors. GO analysis was performed using KOBAS 9.

### PPI network construction

Using STING database to analyze PD-L1 genes and related proteins. The tree diagram of PD-L1 related protein network was constructed with the Cytoscape software. The confidence score cut-off was set to 0.8. The hub scores of the genes in the PPI network were calculated using the cytoHubba app in Cytoscape software. The top nodes were ranked by degrees.

### Using TIMER to produce an inference on the number of tumor-infiltrating immune cells

To systematically analyze the infiltration of immune cells in pancreatic cancer, we employed the TIMER as a comprehensive resource (https://cistrome.shinyapps.io/timer/). We conducted a series of analysis on the expression of PD-L1 in pancreatic cancer and its correlation with the abundance of immune infiltrates. These immune infiltrates include B cells, CD4+ T cells, CD8+ T cells, neutrophils, macrophages, and dendritic cells via gene modules.

### Using MCP-counter to evaluate PD-L1 related immune infiltration

MCP-counter is available R package to estimate the sample immune infiltration 10. From a gene expression matrix, it produces for each individual an available score of cells originating from monocytes (monocytic lineage), endothelial cells, CD3+ T cells, CD8+ T cells, cytotoxic and B lymphocytes, NK cells, myeloid dendritic cells, neutrophils, and fibroblasts. We employed correlation analysis to estimate the correlation between PD-L1 and immune infiltration.

### Mass spectrometry analysis

To identify the binding proteins of PD-L1, ASPC1 cells were transfected with pcDNA3-Flag vector, Flag-tagged PD-L1. Lysates were immunoprecipitated with Flag-agarose, and then binding proteins were eluted with 2% SDS. Immunoprecipitates were digested with trypsin at 37 °C overnight. Peptides were extracted with 50% acetonitrile/5% formic acid, followed by 100% acetonitrile. Peptides were dried to completion and resuspended in 2% acetonitrile/0.1% formic acid. The peptides were subjected to NSI source followed by tandem mass spectrometry (MS/MS) in Q Exactive^TM^ Plus (Thermo) coupled online to the UPLC. GO enrichment analysis was performed using KOBAS.

### Statistical analysis

SPSS 22.0 statistical software and R (https://www.r-project.org/) were used for data analysis. Measurement data were expressed as X ± s, and independent sample t-test and paired t-test were used for comparison between the two groups. Survival analysis was performed by Kaplan-Meier method and log-rank test. Cox proportional hazard regression model was used to analyze the risk factors affecting the prognosis of patients, and the risk ratio (HR) and 95% confidence interval (95%CI) were calculated. P < 0.05 was considered statistically significant. The criteria for GSEA to determine significance enrichment were P < 0.01 and error discovery rate (FDR) < 0.05.

## Results

### Relationship between the expression level of PD-L1 gene and clinicopathological indexes in tumor tissues

**Figure 1:**
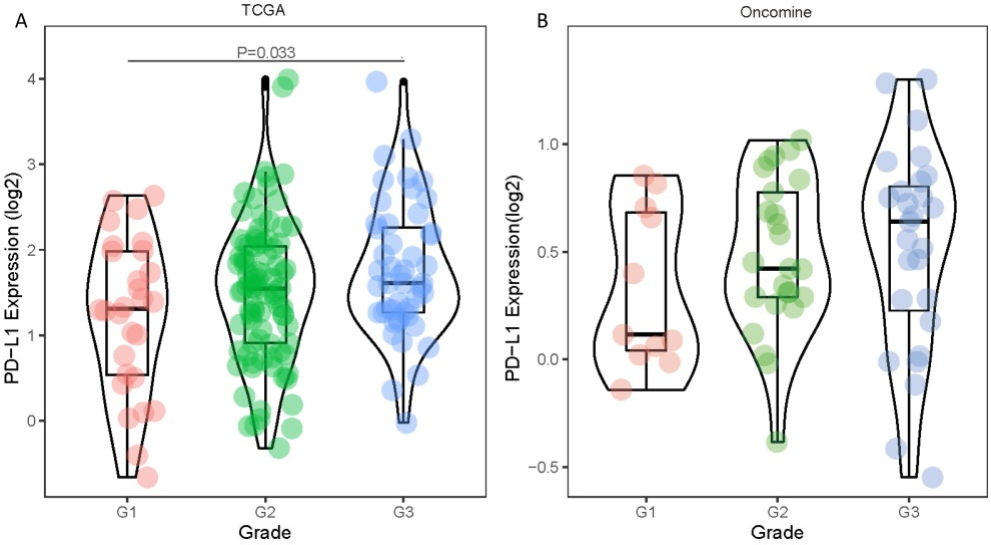
Differences in PD-1 PD-L1 expression between histologic grades. The datasets were from TCGA (**A**) and Oncomine (**B**).

Statistical tabulation analysis of data in TCGA and Oncomine database showed that the expression of PD-L1 was correlated with the pathological grade of tumors. As shown in Figure 1A, by analyzing the TCGA data, we found that the expression of G3 tumors was significantly higher than that of G1 tumors (P=0.033). Similar results were obtained by analyzing the Oncomine datasets. The PD-L1 expression showed an increasing trend with higher pathological grade (Figure 1B). The expression level of PD-L1 gene showed no statistically significant differences between age, gender, pathological stage and TNM stage (all P values were > 0.05) (Table 1).

### Correlation between PD-L1 gene and prognosis of pancreatic cancer patients

**Figure 2:**
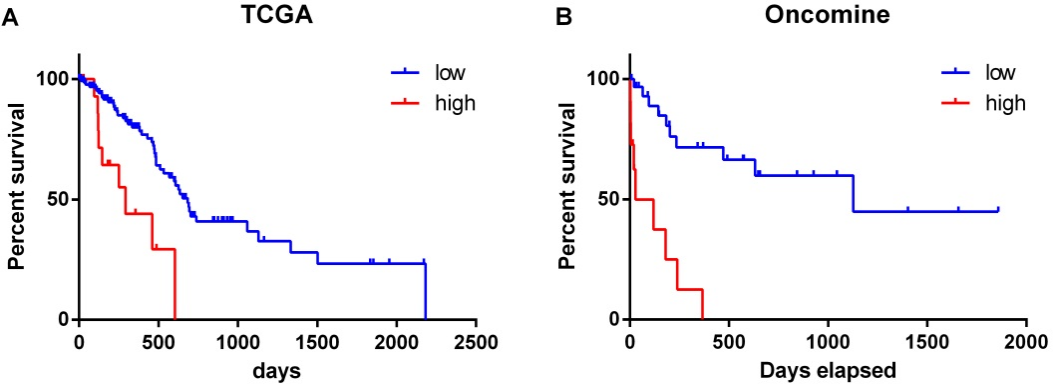
Relationship between the expression level of PD-L1 gene and the survival time of pancreatic cancer patients. The datasets were from TCGA (**A**) and Oncomine (**B**).

The overall survival of patients with high expression of PD-L1 gene was significantly lower than that of patients with low expression (χ^2^ = 12.52, P < 0.001) (Figure 2A). Similar results were obtained by analyzing the Oncomine datasets (χ^2^ = 22.10, P < 0.001) (Figure 2B). These results suggested that high expression of PD-L1 gene is a factor with poor prognosis in pancreatic cancer. Univariate Cox regression analysis suggested that PD-L1 gene expression level, tumor grade and N stage were associated with the overall survival of pancreatic cancer patients (all P < 0.001). Multivariate Cox regression analysis further suggested that the expression level of PD-L1 gene was an independent risk factor affecting the overall survival of pancreatic cancer patients (P < 0. 05) (Table 2).

### Functional enrichment analysis of PD-L1 gene

**Figure 3:**
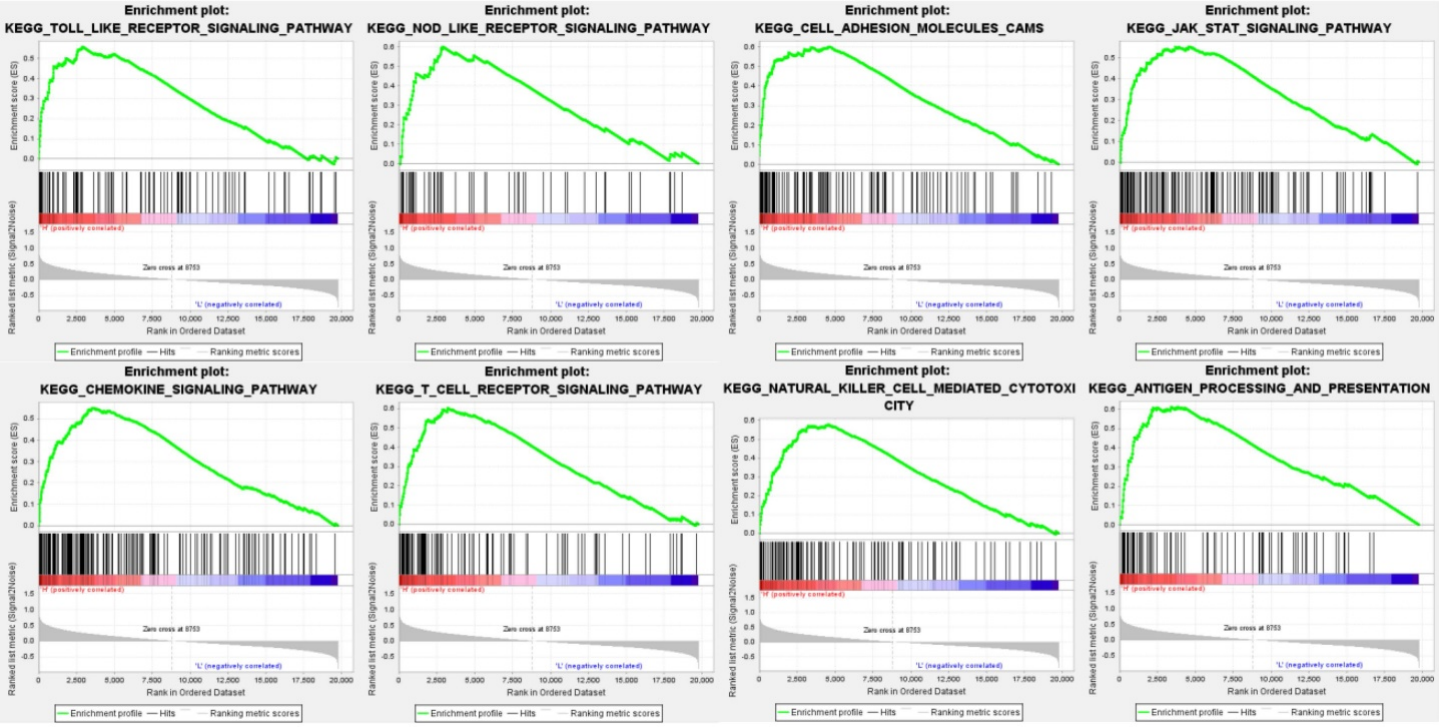
Enrichment plots from gene set enrichment analysis (GSEA) showing differential enrichment of genes in KEGG with high PD-L1 expression.

Data analysis shows that in the TCGA repositories PD sample enrichment - high L1 gene expression to toll-like receptors signaling pathways, NOD receptors signaling pathways, cell adhesion molecules, JAK/STAT signaling pathway, chemokine signaling pathways, T cell receptors signaling pathways, NK cell-mediated cytotoxicity, antigen processing and rendering genetic pathways associated with 8 sets (P < 0. 01, FDR < 0. 05), PD - L1 gene may through these pathways play a role of promoting the development of cancer (Table 3, Figure 3).

### Co-expression analysis of genes associated with PD-L1

**Figure 4:**
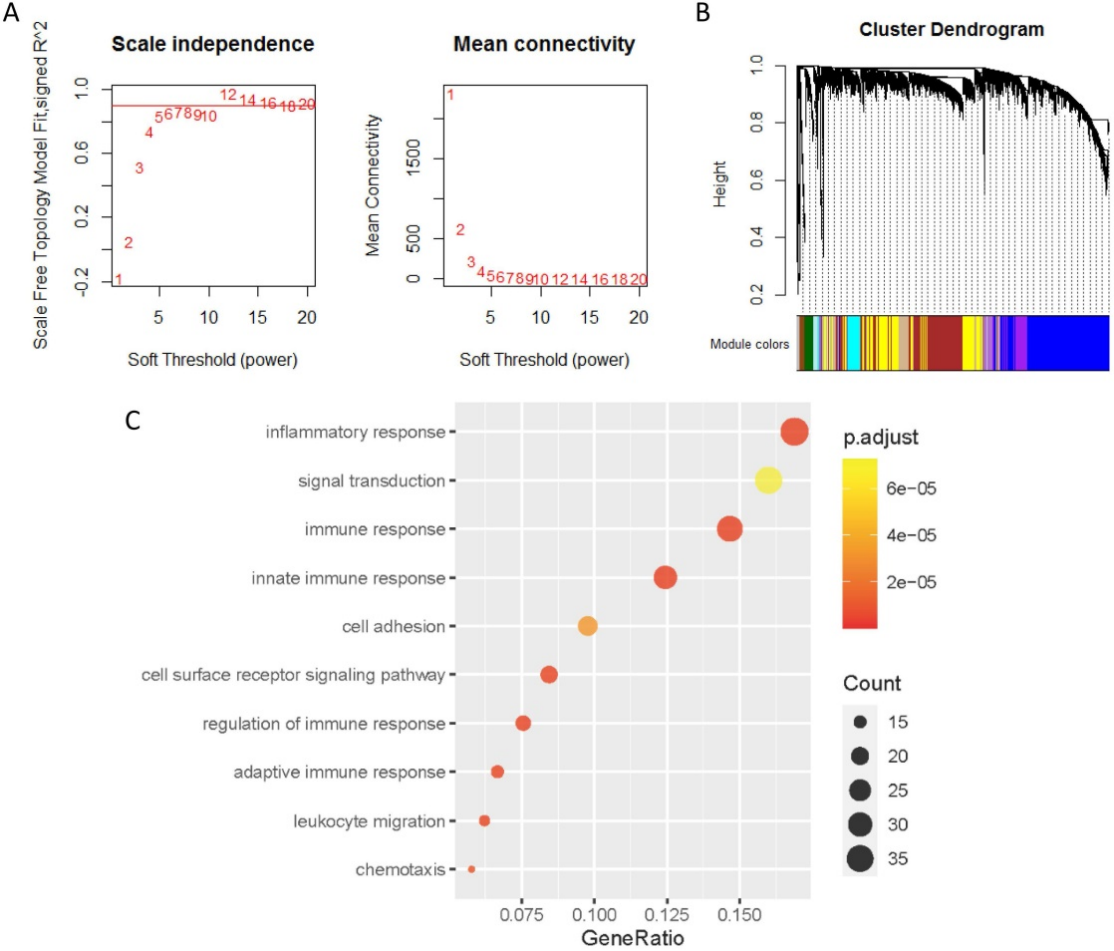
** Co-expression analysis of genes associated with PD-L1.** (**A**) Soft threshold selection in the WGCNA network analysis. (**B**) Gene distribution in the WGCNA network analysis. (**C**) GO analysis for the PD-L1 co-expression genes.

The co-expression genes of PD-L1 were analyzed by WGCNA. When the soft threshold β value is set at 5, the connections between genes are distributed in a scale-free network (Figure 4A). A total of 44 modules were obtained by WGCNA analysis (Figure 4B). PD-L1 belongs to Salmon module. We ended up with 226 genes associated with PD-L1. Then, all 226 PD-L1-related genes were selected for enrichment analysis. PD-L1 correlated genes mainly enriched in biological process of inflammatory response, signal transduction, immune response and cell surface receptor signaling pathway (Figure 4C).

### PD-L1 expression is correlated with immune infiltration level and cumulative survival in pancreatic cancer from TIMER

**Figure 5:**
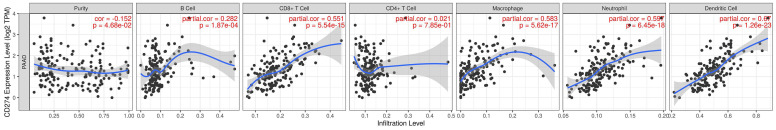
PD-L1 expression level has significant correlations with infiltrating levels of B cell, CD8+ T cells, Macrophages, Neutrophils and Dendritic cells.

The previous analysis suggested that the signaling pathways and interaction molecules related to PD-L1 were mostly immune-related. This leads us to investigate the correlation between PD-L1 expression and immune infiltration in pancreatic cancer. Our results showed the correlation of PD-L1 expression level with poorer prognosis and high immune infiltration in pancreatic cancer. Moreover, a positive correlation exists between the PD-L1 expression level and infiltrating levels of B cell (r = 0.282, P = 1.87e-03), CD8+ T cells (r = 0.551, P = 5.54E-15), Macrophages (r = 0.583, P = 5.62E-17), Neutrophils (r =0.597, P = 6.45E-18), and DCs (r = 0.670, P = 1.26E-23) in pancreatic cancer (Figure 5).

### PD-L1 expression and immune infiltration

**Figure 6:**
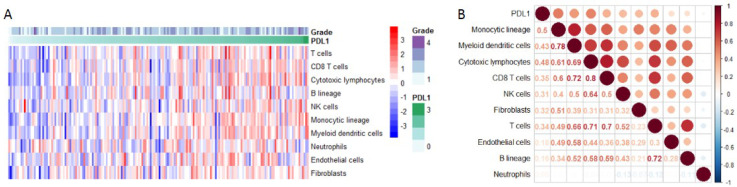
** PD-L1 expression and immune infiltration.** (**A**) The proportion of all immune infiltration components in pancreatic cancer. (**B**) Co-expression analysis between PD-L1 and immune infiltration.

Using Microenvironment Cell Populations-counter, we analyzed the relationship between PD-L1 and immune cell population based on transcriptome data. The results showed that PD-L1 was mainly associated with monocytic lineage (r =0.5) (Figure 6A-B).

### PD-L1 associated PPI network

**Figure 7:**
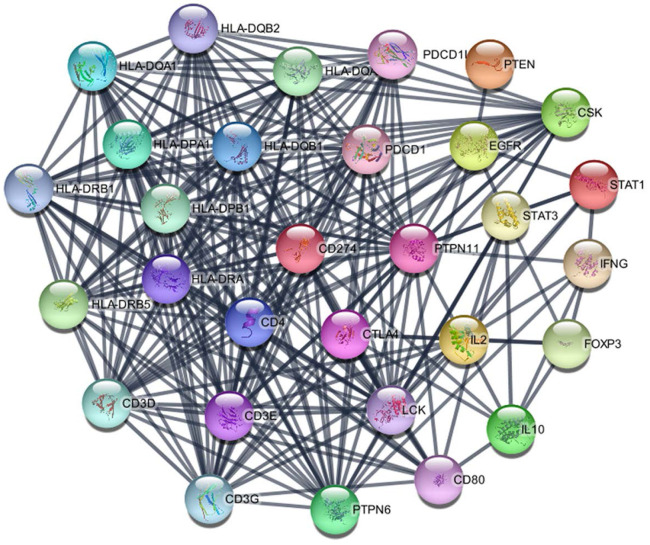
PPI network of PD-L1.

The PD-L1 associated PPI network was established with 31 nodes and 269 edges (Figure 7). The PPI network showed that some genes had close relationship with PD-L1, for instance, PTPN11, LCK, CD3G, CD3D, CD3E, CD4, PTPN6, PDCD1, HLA-DRA and HLA-DRB1. The top 10 hub genes were screened according to their degree values, as shown in Table 4.

### Enrichment analysis of PD-L1 binding proteins

**Figure 8:**
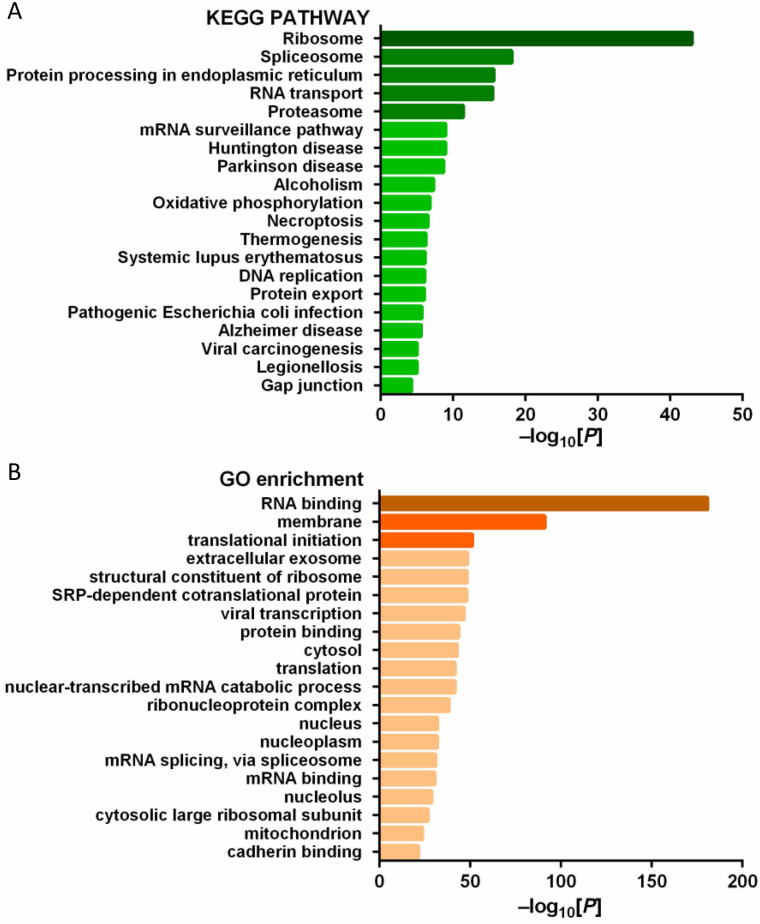
** (A)** KEGG pathway analysis of PD-L1 interacting protein; **(B)** GO gene function analysis of PD-L1 interacting protein.

In order to explore the function of PD-L1 in tumor cells, we studied the PD-L1 interacting protein. A number of potential PD-L1 interacting proteins were identified by immunoprecipitation combined with mass spectrometry analysis of pancreatic cancer cells (Figure 8A-B).

## Discussion

Using data from TCGA, we found that the expression of PD-L1 in G3 pancreatic cancer was significantly higher than that in G1 stage, indicating that PD-L1 was correlated with the malignant degree of pancreatic cancer. By analyzing the correlation between PD-L1 gene expression level and the prognosis of patients with pancreatic cancer, it was found that the overall survival of patients with high PD-L1 gene expression was significantly lower than that of patients with low PD-L1 gene expression. Multivariate Cox regression analysis showed that PD-L1 gene expression level was an independent risk factor affecting the overall survival of patients with pancreatic cancer. These results are consistent with previous studies, suggesting that PD-L1 is expected to be a molecular marker for tumor diagnosis and prognosis of cancer patients 11.

By means of gene set enrichment analysis, this study found that the PD-L1 gene expression group was significantly enriched in eight signaling pathways, including toll-like receptor signaling pathway and NOD-like receptor signaling pathway. We subsequently identified co-expressed genes of PD-L1 by means of WGCNA. A total of 226 genes correlated with PD-L1 were screened. The GO enrichment analysis of PD-L1 correlated genes suggested biological process of inflammatory response, signal transduction, immune response and cell surface receptor signaling pathway. These results suggest that PD-L1 may have two functions, one is immune regulation, the other is signal transduction.

Previous studies have shown that PD-L1 has immune-modulatory function. PD-L1 can inhibit T cell proliferation and cytokine secretion, and negatively regulate lymphocyte activation 12. PD-L1 can be expressed on the surface of tumor 13. By binding to PD-1 on the surface of T cells, it induces apoptosis, incapacity and depletion of T cells, and then inhibits the activation, proliferation and anti-tumor function of tumor antigen-specific CD8+T cells to achieve tumor immune escape 14. According to the correlation analysis of PD-L1 with immune cell populations, we found that PD-L1 were mainly related with monocytic lineage. Data analysis based on TIMER also found a significant correlation between PD-L1 and macrophage infiltration. The interaction of PD-L1 with immune infiltration is a promising research direction in the future.

Many clinical studies have attempted to use PD-L1 mAb for the treatment of a variety of tumors, and found that some tumors expressed PD-L1 highly, but the efficacy of mAb was poor. For example, in a phase II clinical study on immunotherapy of pancreatic cancer, although 12% of patients had high PD-L1 expression (more than 25% of tumor cells expressed PD-L1), However, PD-L1 inhibitor therapy did not achieve ideal objective remission rate 15. Monoclonal drugs can specifically block the binding of PD-L1 on tumor cell membrane to T cell PD-1, but cannot block the action of PD-L1 in tumor cells. The high expression of PD-L1 in pancreatic cancer is not effective with monoclonal antibody treatment, suggesting that PD-L1 has a tumor-promoting regulatory mechanism independent of PD-1. Previous studies mostly focused on the PD-L1/PD-1 axis, believing that the function of PD-L1 is mainly to bind with PD-1 and act on T cells. Due to the short intracellular segment of PD-L1, few scholars have studied PD-L1 mediated signal transduction in tumor cells 6. However, a recent study found that PD-L1 has the function of regulating RNA degradation 16. This is consistent with the results of this study. Using PD-L1 as bait protein, we identified a number of potential PD-L1 interacting proteins through immunoprecipitation combined with mass spectrometry analysis. Because the cells used were pancreatic cancer cell lines without mesenchymal cells, all the interacting proteins identified were expressed by tumor cells. Using KEGG and GO analysis, we found that the genes corresponding to these proteins were enriched in RNA binding, ribosome, spliceosome and other biological processes or pathways. This finding suggests that PD-L1 may have a certain function as a signal molecule, which is worthy of further study.

This study focused on pancreatic cancer, but there are still some potential implications for additional types of cancer. Previous studies have reported that PD-L1 is correlated with the prognosis of breast cancer and colorectal cancer, which is consistent with the results of our study 11, 17. We also found that PD-L1 is associated with immune cell infiltration in the tumor microenvironment, which has been confirmed in head and neck squamous cell carcinoma 18. Finally, we found that PD-L1 binding proteins are enriched in the biological process of RNA binding. This result also coincides with a previous study based on colorectal and breast cancer cells 16.

Although this study provides clues and basis for in-depth discussion of the role of PD-L1 gene in pancreatic cancer, the occurrence and development of tumor is a complex regulatory process coordinated by multiple genes, and the specific mechanism of PD-L1 gene's involvement in the development of pancreatic cancer through the above pathways remains to be further clarified by follow-up studies.

In conclusion, this study revealed that PD-L1 gene is involved in the occurrence and development of pancreatic cancer as a pro-oncogene through the use of relevant bioinformatics methods, which is helpful to improve the monitoring strategy for the recurrence of pancreatic cancer patients after treatment, thus providing a new idea for early diagnosis and early treatment in clinical practice.

## Figures and Tables

**Table 1:** Relationship between the expression level of PD-L1 gene and clinicopathological indicators in patients with pancreatic cancer

Clinical characteristics	n	PD-L1 (mean±sd)	*t* value	*p* value
**Age**			0.817	0.415
≤65	87	3.617±0.244		
>65	82	3.319±0.273		
**Gender**			0.211	0.833
Male	92	3.437±0.263		
Female	77	3.514±0.249		
**Pathologic stage**			0.748	0.456
I/II	162	3.501±0.189		
III/IV	7	2.815±0.491		
**T stage**			1.521	0.130
T1/T2	29	2.865±0.239		
T3/T4	140	3.598±0.213		
**N stage**			0.621	0.536
N0	48	3.292±0.363		
N1	121	3.544±0.211		
**M stage**			0.483	0.629
M0	165	3.486±0.186		
M1	4	2.904±0.703		

**Table 2:** Analysis of single and multiple factors affecting the prognosis of patients with pancreatic cancer

Variables	Univariate analysis		Multivariate analysis	
	HR (95% CI)	*P*-value	HR (95% CI)	*P*-value
PD-L1	3.531 (1.676-7.438)	0.001	3.673 (1.662-8.118)	0.001
age	1.193 (0.706-2.017)	0.510	1.344 (0.761-2.372)	0.308
gender	1.167 (0.684-1.989)	0.571	1.337 (0.763-2.343)	0.310
Pathologic stage	0.734 (0.101-5.338)	0.760	1.249 (0.162-9.607)	1.249
Histologic grade	1.820 (1.062-3.120)	0.029	1.356 (0.755-2.435)	0.307
T stage	2.158 (0.969-4.806)	0.060	1.315 (0.544-3.178)	0.543
N stage	2.344 (1.208-4.547)	0.012	2.024 (0.964-4.247)	0.062

**Table 3:** The pathway gene sets of positive correlation of PD-L1 gene enrichment in pancreatic cancer samples

Gene set name	ES	NES	NOM P-val	FDR
TOLL LIKE RECEPTOR SIGNALING PATHWAY	0.557738	2.085781	0	0.00474
NOD LIKE RECEPTOR SIGNALING PATHWAY	0.601119	2.069144	0	0.00158
CELL ADHESION MOLECULES	0.601111	2.055942	0	0.001383
JAK STAT SIGNALING PATHWAY	0.55201	2.038273	0	0.002334
CHEMOKINE SIGNALING PATHWAY	0.549998	2.020226	0	0.003235
T CELL RECEPTOR SIGNALING PATHWAY	0.603192	1.965347	0	0.007443
NATURAL KILLER CELL MEDIATED CYTOTOXICITY	0.579307	1.900098	0	0.01285
ANTIGEN PROCESSING AND PRESENTATION	0.611949	1.826574	0.01006	0.025689

ES: enrichment score; NES: normalized enrichment score; NOM: nominal; FDR: false discovery rate. Gene sets with NOM p-value < 0.05 and FDR q-value < 0.05 are considered as significant.

**Table 4:** The degree value of the top 10 hub genes

Gene symbol	Gene description	Degree
*PTPN11*	Protein tyrosine phosphatase, non-receptor type 11	26
*LCK*	Lymphocyte cell-specific protein-tyrosine kinase	25
*CD3G*	T-cell surface glycoprotein CD3 gamma chain	22
*CD3D*	T-cell surface glycoprotein CD3 delta chain	22
*CD3E*	T-cell surface antigen T3/Leu-4 epsilon chain	21
*CD4*	T-cell surface antigen T4/Leu-3	21
*PTPN6*	Protein tyrosine phosphatase, non-receptor type 6	21
*PDCD1*	Programmed cell death protein 1	20
*HLA-DRA*	HLA class II histocompatibility antigen, DR alpha chain	20
*HLA-DRB1*	HLA class II histocompatibility antigen, DRB1-15 beta chain	20

## Abbreviations

PD-L1: Programmed cell death-Ligand 1
TCGA: The Cancer Genome Atlas
GSEA: gene set enrichment analysis
WGCNA: Weighted correlation network analysis
KEGG: Kyoto encyclopedia of genes and genomes
GO: Gene ontology
Co-IP: Co-Immunoprecipitation
